# Ophthalmic Artery Morphology and Hemodynamics Associated with White Matter Hyperintensity

**DOI:** 10.7150/ijms.94677

**Published:** 2024-06-11

**Authors:** Xiao-lei Zhang, Xue-ru Cheng, Yan-ling Wang, Ying-xiang Huang, Jia-lin Wang

**Affiliations:** 1 Department of Ophthalmology, Beijing Friendship Hospital, Capital Medical University, Beijing 100050, China.; 2 Institute of Ophthalmology, Capital Medical University, Beijing, China.

**Keywords:** white matter hyperintensity, ophthalmic artery, hemodynamics, computational fluid dynamics

## Abstract

**Purpose:** To investigate morphological and hemodynamic characteristics of the ophthalmic artery (OA) in patients with white matter hyperintensity (WMH), and the association of the presence and severity of WMH with OA characteristics.

**Methods:** This cross-sectional study included 44 eyes of 25 patients with WMH and 38 eyes of 19 controls. The Fazekas scale was adopted as criteria for evaluating the severity of white matter hyperintensities. The morphological characteristics of the OA were measured on the basis of three-dimensional reconstruction. The hemodynamic parameters of the OA were calculated using computational fluid dynamics simulations.

**Results:** Compared with the control group, the diameter (16.0±0.27 mm vs. 1.71±0.18 mm, *P*=0.029), median blood flow velocity (0.12 m/s vs. 0.22 m/s, *P*<0.001), mass flow ratio (2.16% vs. 3.94%, *P*=0.012) and wall shear stress (2.65 Pa vs. 9.31 Pa, *P*<0.001) of the OA in patients with WMH were significantly decreased. After adjusting for confounding factors, the diameter, blood flow velocity, wall shear stress, and mass flow ratio of the OA were significantly associated with the presence of WMH. Male sex and high low-density protein level were associated with moderate-to-severe total WMH, and smoking was associated with the moderate-to-severe periventricular WMH.

**Conclusions:** The diameter, blood flow velocity, mass flow ratio, and wall shear stress of the OA were independently associated with the presence of WMH. Atherosclerosis might be involved in the common mechanism of the occurrence of WMH and the OA changes.

## Introduction

White matter hyperintensity (WMH) is one of typical manifestations of cerebral small vessel disease (CSVD), which is common in the elderly population and is considered an early marker of brain frailty 1. Studies have reported that WMH increases the risk of ischemic and hemorrhagic stroke, cognitive decline, dementia, depression, and mortality 2.

Patients with WMH undergo demyelination, axonal damage, loss of oligodendrocytes, and impaired white matter integrity 3. The lesions are usually scattered in the deep or periventricular white matter, manifested as high signal patchy areas on the T2-weighted or flair-attenuated inversion recovery (FLAIR) sequences on magnetic resonance imaging (MRI). A scoring system based on MRI has been proposed to evaluate the burden of CSVD 4, among which the Fazekas score is used to evaluate the burden of WMH according to the presence, location, and range of lesions 5. Although it is a subjective visual assessment, Fazekas score is convenient in primary healthcare services, and the high correlation between the Fazekas score and volumetric assessment of WMH has previously been confirmed 6.

Studies have shown a correlation between WMH and changes in retinal blood vessels and ocular ischemia 7, 8. There might be common mechanisms in the occurrence of WMH and retinal vascular changes, because of the similar characteristics of retinal arteries and cerebral arterioles, including similar diameters, embryologic origin, and blood barriers. However, compared with retinal arteries, the ophthalmic artery (OA) was thought to more directly and earlier reflect the changes of the ocular blood flow 9. Our previous study showed that the presence of WMH was correlated with the OA blood flow velocity 10. Therefore, exploring the correlation between characteristics of the OA and WMH may be important for early detection of ocular or cerebral small vessel diseases.

Computed tomography angiography (CTA) is a non-invasive method for visualization of the OA, which can clearly display the vascular morphology and complex course of the OA 11. On the basis of CTA images, a three-dimensional (3D) model can be reconstructed and the morphological parameters of the OA can be measured using the computer software. Besides, the hemodynamic characteristics of the OA can be obtained through computational fluid dynamics (CFD) simulation. These parameters can reflect the actual state of the OA in the body.

This study aimed to investigate the OA morphological and hemodynamic characteristics in patients with WMH, and the correlation of WMH with the characteristics of the OA and clinical characteristics. We hypothesized that the decrease in the diameter and blood flow velocity of the OA would be associated with the presence of WMH.

## Methods

### Patients and data collection

The study population of this cross-sectional study consisted of 44 consecutive patients admitted to Beijing Friendship Hospital from November 2019 to January 2023 who underwent head and neck CTA and brain MRI for various reasons. The medical records of all participants were reviewed. This study was performed in accordance with the principles outlined in the Declaration of Helsinki. This study was approved by the Ethics Committee of Beijing Friendship Hospital, Capital Medical University (2020-P2-008), and informed consent was obtained from each participant. The MRI images were carefully examined, and the burden of WMH was semiquantitatively measured based on FLAIR and T2-weighted images using Fazekas visual grading scale. A total score ranging from 0 to 6 was the sum of Fazekas scores of periventricular and deep WMHs. Patients with the total WMH score of ≥ 1 were included in the WMH group, while patients with a total WMH score of 0 and no other signs of CSVD were included in the control group. The WMH group and control group were age- and sex- matched. The exclusion criteria for all participants were as follows: (1) no initial OA imaging visualized clearly on CTA images; (2) any cerebrovascular accident influencing the evaluation of WMH or other CSVD signs on MRI, including acute massive cerebral infarction or cerebral hemorrhage; (3) brain tumors.

### Brain MRI acquisition and analysis

All participants underwent brain MRI scans using a GE Discovery MR750 3.0 T MRI scanner (GE Healthcare, Waukesha, WI, USA). The sequences included T1-weighted imaging, T2-weighted imaging, FLAIR, diffusion-weighted imaging, and susceptibility-weighted imaging (SWI).

**Figure 1:**
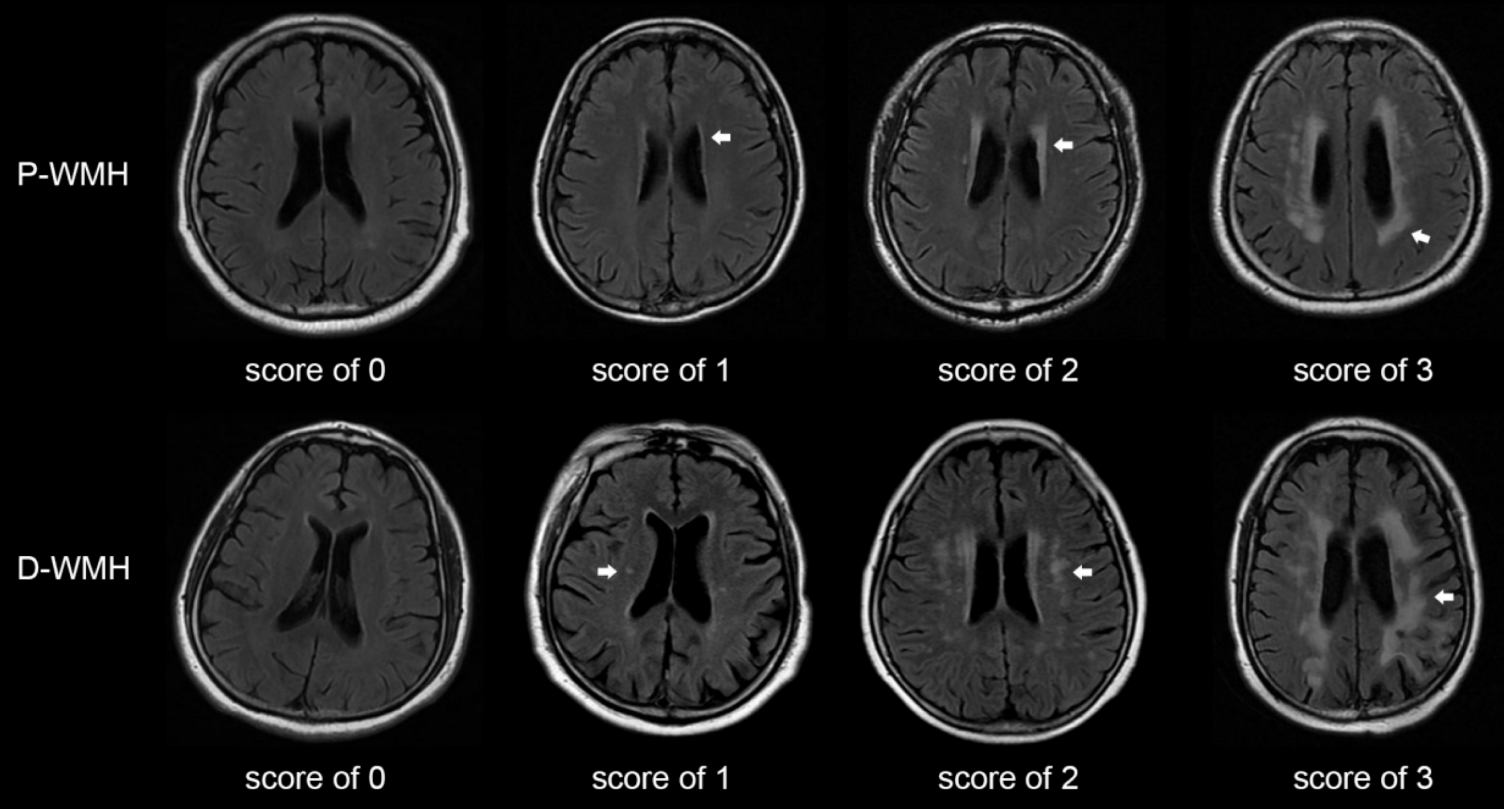
Score of periventricular and deep WMH using the Fazekas scale. Periventricular WMH is graded as follows (arrow): 0=absence, 1=caps or pencil-thin lining, 2=smooth halo, and 3=irregular periventricular WMH extending into the deep white matter. Deep WMH is graded as follows (arrow): 0=absence, 1=punctuate foci, 2=beginning confluence of foci, and 3=large confluent areas. P-WMH, periventricular WMH; D-WMH, deep WMH.

The Fazekas scale was adopted as the criterion for evaluating the severity of WMH 5. The total WMH score was rated on an ordinal scale from 0 to 6, according to the presence, location, and range of WMH lesions on MRI. The lesions were divided into periventricular WMH (P-WMH) and deep WMH (D-WMH) according to the location, with a score of 0-3 for each location (Fig. 1). The rating criteria have been previously published 12-14. Patients with WMH were divided into mild WMH group (total Fazekas score of 1-2) and moderate-to-severe WMH group (total Fazekas score of 3-6). Patients with P-WMH or D-WMH were respectively categorized as none/mild group (periventricular or deep Fazekas score of 0-1) and moderate-to-severe group (periventricular or deep Fazekas score of 2-3). Other CSVD signs were assessed according to the rating criteria described by Wang *et al.* 10.

### CTA data acquisition and 3D reconstruction

CTA examination was performed by using a 64-row multidetector computed tomography scanner (LightSpeed VCT; GE Healthcare, Chicago, IL, USA), extending from the aortic arch to the skull base. The detailed scanning parameters were as follows: reconstruction interval, 0.625 mm; matrix, 512 × 512; layer spacing, 0.8 mm. Based on the CTA images, the 3D model of the OA was reconstructed in Mimics 21.0 (Materialise, Ann Arbor, MI, USA). The OA was divided into intracranial, intracanalicular, and intraorbital segments 15. The centerline best-fit diameter of the initial part of the OA (corresponding to the intracranial segment) and the angle between the OA and ipsilateral internal carotid artery were measured in the Mimics software.

### CFD simulation

Before the CFD preprocessing, Geomagic Studio 14.0 (3D Systems, Rock Hill, SC, USA) was used to smoothen the model surface, obtaining a stereolithographic 3D model of the vessel segment. Then the stereolithographic 3D model was further processed in the Integrated Computer Engineering and Manufacturing code for Computational Fluid Dynamics (ICEM CFD). Each model was discretized into approximately 0.3 million tetrahedron and triangular prism mixed elements. ANSYS Fluent 19.0 (ANSYS, Inc., Canonsburg, PA, USA) was used for hemodynamic simulation calculation, with a finite-volume method. The numerical simulation was based on the Navier-Stokes equation and mass conservation equation:

ρ(u^→^ ⋅∇)u^→^ +∇p-μΔu^→^ =0, (1)

∇⋅u^→^ =0, (2)

where ρ is blood density, u is velocity vector, p is pressure, and μ is blood viscosity. Blood in the simulation was treated as a Newtonian fluid with constant viscosity (µ = 3.5 × 10^-3^ kg/ms, ρ = 1050 kg/m^3^). The artery wall was considered rigid and no slip. The boundary conditions were defined with a constant inlet velocity of 0.34 m/s (which is the blood velocity at the internal carotid artery siphon, based on the setting of Kojima *et al.*) 16 and a pressure boundary condition of 0 Pa at the outlet. The calculation ended when the fluid model converged. The hemodynamic parameters including the blood flow velocity, wall shear stress, and pressure of the OA were measured in the initial part of the OA (corresponding to the intracranial segment), and the mass flow ratio of the OA to the ipsilateral internal carotid artery was calculated.

### Statistical analyses

Data normality was tested. Data of normal continuous variables were presented as mean ± standard deviation, and skewed variables were presented as median (25th percentile, 75th percentile). Categorical data were presented as numbers (percentages). According to the data normality, differences in continuous variables were evaluated using the independent sample t-test or the Mann-Whitney U test. Fisher's exact test or the χ2 test was used to analyze the categorical variables. The univariate logistic regression analysis and multivariate logistic regression analysis (forward: conditional) were performed to explore the correlation between the presence or severity of WMH and the OA characteristics. Values of the OA blood flow velocity were transformed into natural logarithms in the logistic regression analysis. *P*<0.05 indicated statistical significance.

## Results

### Patient baseline clinical characteristics

This study included 44 eyes of 25 patients with WMH (median age, 65 years; 68.2% male) and 38 eyes of 19 controls (median age, 63 years; 71.1% male). Table 1 presents the baseline data of all participants. The differences in hypertension (75.0% vs. 52.6%, *P*=0.035), systolic blood pressure (SBP; median SBP, 146.00 mmHg vs.128.00 mmHg, *P*<0.001) and diastolic blood pressure (DBP; median DBP, 84.00 mmHg vs.76.00 mmHg, *P*=0.001) between patients with WMH and the control group were significant. The median hemoglobin A1c (HbA1c) of patients with WMH was significantly higher than that of the control group (5.68% vs. 5.40%, *P*=0.008). There were no significant differences in other systemic or laboratory parameters between the WMH group and control group (Table 1).

### Morphological and hemodynamic comparison of the OA

**Figure 2:**
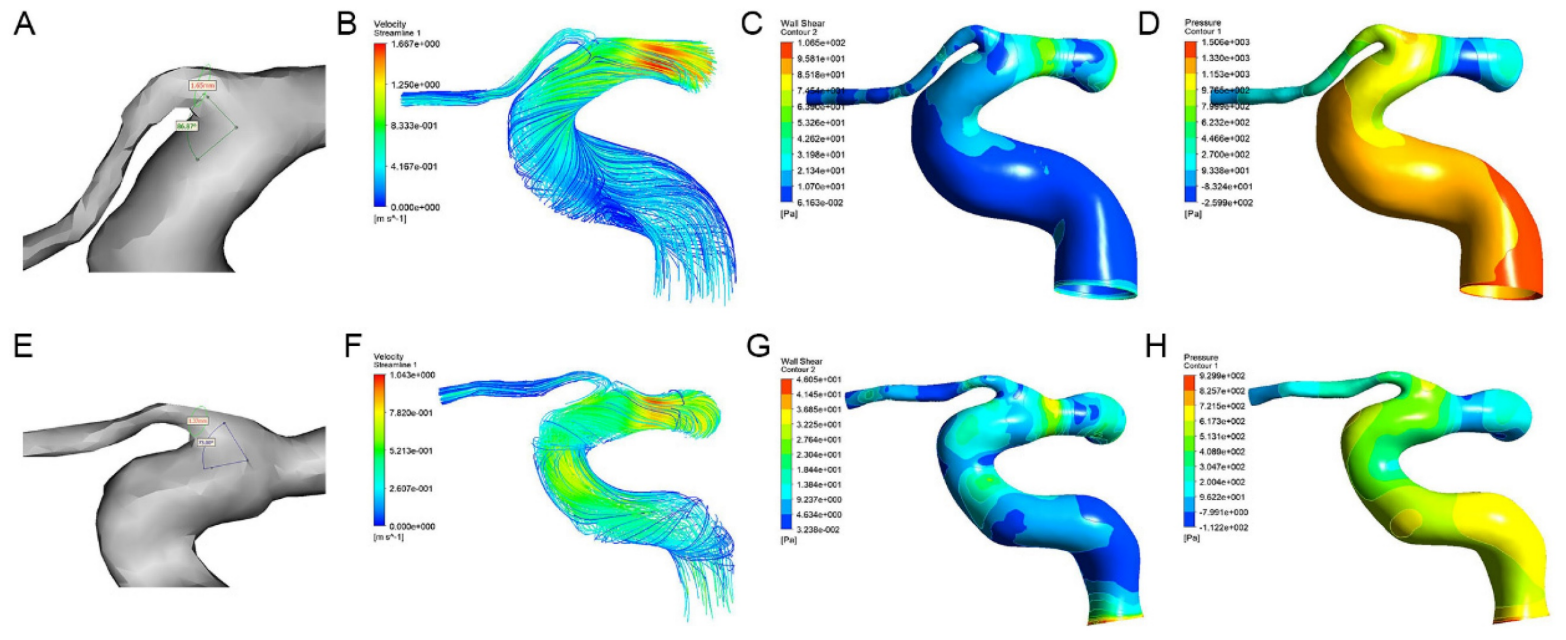
The morphological measurements, blood flow streamlines, wall shear stress contours, and pressure contours of the OA in control group (A-D) and WMH group (E-H). (A) The OA diameter was 1.65mm and the angle was 86.87° in the patient with WMH. (E) The OA diameter was 1.37mm and the angle was 73.00° in the control group. (B-D, F-H) The color indicates the numerical values of the blood flow velocity, wall shear stress, and pressure.

The morphological and hemodynamic measurements of the OA were shown in Figure 2. The mean OA diameter in WMH was significantly smaller than that in the control group (1.60±0.27 mm vs. 1.71±0.18 mm, *P*=0.029). The median blood flow velocity of the OA in WMH was significantly lower than that in the control group (0.12 m/s vs. 0.22 m/s, *P*<0.001). Compared with the control group, the median mass flow ratio of the OA to the ipsilateral internal carotid artery in patients with WMH was significantly reduced (2.16% vs. 3.94%, *P*=0.012). The wall shear stress of the OA in WMH was significantly lower than that in the control group (2.65 Pa vs. 9.31 Pa, *P*<0.001). No significant difference was found in the angle and the pressure of the OA (Table 2).

### Correlation of the presence of WMH with the OA characteristics and clinical parameters

In the multivariate logistic regression analysis, the diameter (odds ratio [OR], 0.028; 95% confidence interval [CI], 0.001-0.730; *P*=0.032), blood flow velocity (OR, 0.099; 95% CI, 0.024-0.400; *P*=0.001), wall shear stress (OR, 0.811; 95% CI, 0.708-0.928; *P*=0.002), and mass flow ratio (OR, 0.754; 95% CI, 0.615-0.924; *P*=0.007) of the OA were significantly associated with the presence of WMH after adjusting for age, sex, SBP, DBP, and HbA1c. Besides, SBP and DBP were also independently associated with the presence of WMH (Table 3).

### Correlation of the severity of WMH with clinical characteristics and the OA characteristics

Patients with WMH were divided into mild WMH group (total Fazekas score of 1-2) and moderate-to-severe WMH group (total Fazekas score of 3-6). Patients with P-WMH or D-WMH were respectively categorized as none/mild group (periventricular or deep Fazekas score of 0-1) or moderate-to-severe group (periventricular or deep Fazekas score of 2-3). In the univariate logistic regression analysis, low mass flow ratio and low wall shear stress of the OA were correlated with the moderate-to-severe total WMH and moderate-to-severe P-WMH (Table 4). However, after adjusting for sex, smoking, total cholesterol, and low-density lipoprotein (LDL), these correlations were no longer significant. Male sex and decreased LDL were associated with moderate-to-severe total WMH, and smoking was associated with moderate-to-severe P-WMH. No correlation was found between all OA characteristics and the severity of D-WMH (all *P*>0.05). In addition, there was no significant correlation between the OA characteristics and the total WMH score (all *P*>0.05).

## Discussion

Our study suggested that the diameter and blood flow velocity of the OA were decreased in patients with WMH. One possible cause is that atherosclerosis plays a role in the pathogenesis of WMH and OA changes. Atherosclerosis could cause carotid artery stenosis, which leads to chronic hypoperfusion of terminal small vessels, inducing ocular and brain parenchymal ischemic lesions 17, 18. The endothelial dysfunction caused by oxidative stress and inflammation in atherosclerosis may be the pathological mechanisms of decreased OA diameter 19. Besides, the resistance of the OA was increased in patients with atherosclerotic diseases, which may be may be the reason for decreased blood flow velocity of the OA 20.

Our results showed a significant decrease in the wall shear stress of the OA in the WMH group compared to that in the control group. This might be due to a decrease in OA blood flow in the WMH group. According to Poiseuille's law, the wall shear stress decreases due to a severe decrease in blood flow 21. In addition, low wall shear stress might mediate arterial wall remodeling by inducing endothelial cells to transform into atherosclerotic endothelial phenotype 22, increasing the accumulation of low-density lipoproteins in the intima of the arterial wall 23 and enhancing the expression of proinflammatory genes 24.

These changes could cause vascular dysfunction and stenosis, and may further cause a decrease in the OA diameter.

Ischemia and hypoperfusion may be the pathogenesis of WMH 25, 26. Perfusion disturbances may predate microstructural alterations and visible lesions 27. Prolonged low blood flow affects small deep perforating arteries, and severe local hypoperfusion may lead to mild brain injury, manifested as WMH 28. The white matter receives the blood supply from the microvascular network in the pia mater, which is formed by the anastomosis of cortical artery branches from the anterior cerebral artery, middle cerebral artery, and posterior cerebral artery. The periventricular white matter mainly receives blood supply from the lenticulostriate arteries (originating from the middle cerebral artery) and the anterior choroidal artery (originating from the internal carotid artery). Both the anterior cerebral artery and middle cerebral artery originate from the internal carotid artery. Therefore, white matter lesions may be related to stenosis and decreased blood flow of the internal carotid artery. The OA also originates from the internal carotid artery. Stenosis of the internal carotid artery can cause a decrease in perfusion of both the eyes and brain. Decreased blood flow in OA may lead to a decrease in wall shear stress and further reduction in diameter, which explains the correlation between OA characteristics and WMH.

Although there are similarities between cerebral small vessels and retinal vessels, including the similar diameters, embryologic origin, and blood barriers, the OA is the main blood supply to the eyes and can more directly and earlier reflect the changes of the ocular blood flow compared with retinal arteries 9. Blixt *et al.* found that the vasoconstriction of the OA increased 48 hours after cerebral ischemia, whereas the retinal damage occurred 72 hours after ischemia 29. This finding suggested that the OA reflects changes in ocular hemodynamics earlier than retinal vessels after cerebral ischemia. Therefore, the OA may reflect changes in ocular blood flow in patients with WMH earlier than the retinal vascular system.

Among various representative biomarkers in CSVD, lacunar infarction and cerebral microbleeds are mainly caused by occlusion or rupture of a single perforating artery or arteriole, while WMH is more diffusely distributed and observable in the early stages of CSVD 30. Focusing on WMH may have clinical importance. A previous study has found that patients with WMH had significantly thinner Henle fiber layers, outer nuclear layers and photoreceptor outer segments than healthy controls 31. Ulusoy *et al.* found deeper foveal vessel density and superior hemisphere vessel density in patients with WMH were lower than those in people without WMH using macular optical coherence tomography angiography, indicating an association between WMH and ocular ischemia 8. Qu *et al.* found a significant correlation of the retinal nerve fiber layer, ganglion cell layer, and inner plexiform layer of all participants with the Fazekas score of WMH, after adjustment of age and other confounding factors 12. These might be related to specific regions of the brain affected by white matter lesions. The damage to these regions might cause damage to the connections involving the visual tract, leading to degeneration of the optic nerve and resulting in the changes of these sublayer 32. Conversely, cell death in the retinal ganglion region leads to changes in the retinal nerve fiber layer and may further lead to white matter lesions in the brain covering the visual tract responsible for vision 33. These indicated that patients with WMH might have poor visual prognosis 34, which prompted ophthalmologists to pay attention to the presence and progression of WMH in patients with CSVD.

In addition, we found that SBP and HBA1c were independently correlated with the presence of WMH. Hypertension might cause damage to the automatic regulation of cerebral blood flow, leading to a decrease in cerebral blood flow and the occurrence of WMH. The HbA1c level is a more accurate indicator of average glucose levels, compared with single glucose measurement. Schneider *et al.* found that compared with participants without diabetes and HbA1c <5.7%, those with diabetes and HbA1c ≥7.0% had an increased burden of WMH 35. This might be associated with the increase of blood-brain barrier permeability caused by diabetes 36. Diabetes affects the transfer of glucose and insulin through the blood-brain barrier 37, thereby affecting regional metabolism and microcirculation. In addition, diabetes could also cause damage to the blood-brain barrier and brain microvascular damage through the inflammatory reaction 38. In addition, our results showed that male sex and decreased LDL were associated with moderate-to-severe total WMH, and smoking was associated with moderate-to-severe P-WMH. Staals *et al.* found that male sex and smoking were significantly and independently associated with the total CSVD score 4. Schilling *et al.* found that increasing LDL was associated with a decreased frequency and severity of all MRI markers of CSVD 39. Power *et al.* reported that increasing pack-years of smoking was associated with greater risk of WMH progression 40. These supported our findings.

This study had several limitations. The sample size was small. Second, we adopted the same boundary conditions for all models in the CFD simulation. In the future, more studies on the association between OA changes and WMH are needed using more convenient methods.

In conclusion, the diameter, blood flow velocity, mass flow ratio, and wall shear stress of the OA were independently associated with the presence of WMH. Male sex and decreased LDL were associated with moderate-to-severe total WMH, and smoking was associated with the moderate-to-severe P-WMH. Atherosclerosis might be involved in the common mechanisms of the occurrence of WMH and OA changes.

## Figures and Tables

**Table 1:** Baseline characteristics of patients with WMH

Characteristics	WMH (n=44)	Control (n=38)	*P*
Systemic parameters			
Age, y	65.00 (61.00, 68.00)	63.00 (56.00, 68.25)	0.144
Male	30 (68.2)	27 (71.1)	0.778
Hypertension	33 (75.0)	20 (52.6)	0.035^*^
Diabetes mellitus	21 (47.7)	13 (34.2)	0.215
Dyslipidemia	32 (72.7)	27 (71.1)	0.866
Ischemic heart disease	12 (27.3)	4 (10.5)	0.056
Smoking	21 (47.7)	14 (36.8)	0.320
Laboratory parameters			
SBP, mmHg	146.00 (134.00, 164.00)	128.00 (120.00, 136.00)	<0.001^*^
DBP, mmHg	84.00 (75.75, 88.00)	76.00 (71.00, 79.25)	0.001^*^
TC, mmol/L	4.08 ± 0.91	3.93 ± 1.10	0.509
Triacylglycerol, mmol/L	1.19 (1.02, 1.36)	1.30 (0.96, 1.63)	0.413
LDL, mmol/L	2.32 (1.82, 2.79)	2.21 (1.88, 2.89)	0.703
HBA1c, %	5.68 (5.23, 6.50)	5.40 (5.20, 5.70)	0.008^*^
Homocysteine, μmol/L	12.20 (8.00, 23.23)	12.25 (11.20, 14.00)	0.818

Data are presented as mean ± standard deviation, number (%), or median (25th percentile, 75th percentile). SBP, systolic blood pressure; DBP, diastolic blood pressure; TC, total cholesterol; LDL, low-density protein; HBA1c, hemoglobin A1c. ^*^P < 0.05 is significant.

**Table 2:** The OA characteristics of patients with WMH

OA Characteristics	WMH (n=44)	Control (n=38)	*P*
Diameter, mm	1.60 ± 0.27	1.71 ± 0.18	0.029^*^
Angle, °	74.79 (69.70, 79.75)	75.23 (61.18, 85.32)	0.944
Velocity, m/s	0.12 (0.07, 0.20)	0.22 (0.16, 0.31)	<0.001^*^
Mass flow ratio, %	2.16 (1.17, 4.25)	3.94 (2.20, 6.57)	0.012^*^
Wall shear stress, Pa	2.65 (1.08, 6.98)	9.31 (3.24, 17.45)	<0.001^*^
Pressure, Pa	389.30 (258.75, 544.80)	367.10 (270.24, 598.57)	0.746

Data are presented as mean ± standard deviation or median (25th percentile, 75th percentile). ^*^P < 0.05 is significant.

**Table 3:** Correlation of the presence of WMH with OA characteristics and clinical parameters

Variables	Univariate			Multivariate^a^	
	OR (95% CI)	*P*		OR (95% CI)	*P*
Clinical parameters					
SBP	1.090 (1.045, 1.137)	<0.001^*^		1.116 (1.057, 1.178)	<0.001^*^
DBP	1.073 (1.021, 1.128)	0.005^*^			
HBA1c	1.523 (1.066, 2.176)	0.021^*^		1.634 (1.117, 2.391)	0.011^*^
Characteristics of the OA					
Diameter	0.112 (0.015, 0.854)	0.035^*^		0.028 (0.001, 0.730)	0.032^*^
Angle	1.008 (0.972, 1.047)	0.657			
Velocity^b^	0.139 (0.051, 0.383)	<0.001^*^		0.099 (0.024, 0.400)	0.001^*^
Mass flow ratio	0.844 (0.725, 0.982)	0.029^*^		0.754 (0.615, 0.924)	0.007^*^
Wall shear stress	0.847 (0.767, 0.936)	0.001^*^		0.811 (0.708, 0.928)	0.002^*^
Pressure	1.000 (0.998, 1.001)	0.702			

SBP, systolic blood pressure; DBP, diastolic blood pressure; HBA1c, hemoglobin A1c; OA, ophthalmic artery. ^*^P < 0.05 is significant. ^a^Data are adjusted for age, sex, SBP, DBP, and HbA1c in the logistic regression analysis (forward: conditional). ^b^Variables are transformed to natural logarithms.

**Table 4:** Correlation of the moderate-to-severe WMH with clinical characteristics and OA characteristics

	Variables^a^	Univariate			Multivariate^b^	
		OR (95% CI)	*P*		OR (95% CI)	*P*
Total WMH	Clinical characteristics					
	Female	0.100 (0.023, 0.433)	0.002		0.072 (0.010, 0.522)	0.009
	Smoking	14.778 (2.753, 79.332)	0.002			
	TC	0.158 (0.049, 0.506)	0.002			
	LDL	0.041 (0.005, 0.310)	0.002		0.025 (0.002, 0.342)	0.006
	Characteristics of the OA					
	Mass flow ratio	0.755 (0.590, 0.968)	0.027			
	Wall shear stress	0.833 (0.695, 0.997)	0.047			
						
P-WMH	Clinical characteristics					
	Female	0.139 (0.034, 0.570)	0.006			
	Smoking	12.350 (2.315, 65.874)	0.003		10.450 (1.928, 56.637)	0.007
	TC	0.308 (0.126, 0.753)	0.010			
	LDL	0.158 (0.040, 0.626)	0.009			
	Characteristics of the OA					
	Angle	1.078 (1.002, 1.159)	0.043			
	Mass flow ratio	0.756 (0.592, 0.967)	0.026			
	Wall shear stress	0.833 (0.695, 0.997)	0.047			

WMH, white matter hyperintensity; PV-WMH, periventricular white matter hyperintensity; TC, total cholesterol; LDL, low-density protein; OA, ophthalmic artery. ^a^Variables with *P*<0.05 in the univariate logistic regression analysis are listed. ^b^Data are adjusted for sex, smoking, TC, and LDL in the logistic regression analysis (forward: conditional).

## Abbreviations

WMH: White matter hyperintensity
CSVD: Cerebral small vessel disease
FLAIR: Fluid-attenuated inversion recovery
MRI: Magnetic resonance imaging
OA: Ophthalmic artery
CTA: Computed tomographic angiography
CFD: Computational fluid dynamics
SWI: Susceptibility-weighted imaging
P-WMH: Periventricular white matter hyperintensity
D-WMH: Deep white matter hyperintensity
SBP: Systolic blood pressure
DBP: Diastolic blood pressure
HbA1c: Hemoglobin A1c
LDL: Low-density lipoprotein
